# The effect of engineered disulfide bonds on the stability of *Drosophila melanogaster *acetylcholinesterase

**DOI:** 10.1186/1471-2091-7-12

**Published:** 2006-04-16

**Authors:** Omid Ranaei Siadat, Andrée Lougarre, Lucille Lamouroux, Caroline Ladurantie, Didier Fournier

**Affiliations:** 1 IPBS-CNRS 205 route de Narbonne, Toulouse, France; 2 New Ideas Research Group (NIRG), #11, Proshat Alley, Motahhari Street, Tehran, Iran

## Abstract

**Background:**

Acetylcholinesterase is irreversibly inhibited by organophosphate and carbamate insecticides allowing its use in biosensors for detection of these insecticides. *Drosophila *acetylcholinesterase is the most sensitive enzyme known and has been improved by *in vitro *mutagenesis. However, its stability has to be improved for extensive utilization.

**Results:**

To create a disulfide bond that could increase the stability of the *Drosophila melanogaster *acetylcholinesterase, we selected seven positions taking into account first the distance between Cβ of two residues, in which newly introduced cysteines will form the new disulfide bond and second the conservation of the residues in the cholinesterase family. Most disulfide bonds tested did not increase and even decreased the stability of the protein. However, one engineered disulfide bridge, I327C/D375C showed significant stability increase toward denaturation by temperature (170 fold at 50°C), urea, organic solvent and provided resistance to protease degradation. The new disulfide bridge links the N-terminal domain (first 356 aa) to the C-terminal domain. The quantities produced by this mutant were the same as in wild-type flies.

**Conclusion:**

Addition of a disulfide bridge may either stabilize or unstabilize proteins. One bond out of the 7 tested provided significant stabilisation.

## Background

Acetylcholinesterase (AChE, EC 3.1.1.7) is a serine hydrolase, which catalyzes the hydrolysis of acetylcholine. This enzyme is the target of organophosphate and carbamate insecticides which phosphorylate or carbamoylate the serine of the active site blocking the hydrolysis of the neurotransmitter acetylcholine. The post-synaptic membrane then remains depolarized and synaptic transmission cannot take place so the insect dies. These compounds are used to control proliferation of various agricultural pests: insects, acari and nematodes. One of the consequences is that pesticide residues remain in the environment and are potentially toxic for all animals, including humans since cholinergic transmission is well conserved. Insecticide residues can be detected with biosensors using AChE as biological element to detect low levels of contaminants in crops, soil, water or food samples [1,2].

Drosophila AChE (*Dm*AChE) was found to be the most sensitive enzyme when compared to enzymes of non-insect origin and *in-vitro*-mutagenesis has permitted the selection of enzymes up to 300-fold more sensitive [3,4]. But like most enzymes from mesophilic organisms, *Dm*AChE is not stable, and this instability precludes its utilization in biosensors. It can be stabilized by additives: proteins such as bovine serum albumin, reversible inhibitor, polyethylene glycol or by encapsulation in liposomes [5-8]. Another way to stabilize the enzyme is to use *in vitro *mutagenesis to modify the primary structure of the protein. Elimination of a free cysteine and mutation of the hydrophobic residues at the protein surface into hydrophilic residues have been used to increase the stability of *Dm*AChE [9,10]. Here we focused on another method: engineering new disulfide bridges.

Disulfide bonds are present in most extracellular proteins, where they presumably stabilize the native conformation by lowering the entropy of the unfolded form [11] or by decreasing the unfolding rate of irreversibly denatured proteins [12,13]. This stabilizing property makes disulfide bond cross-linking an attractive strategy for engineering additional conformational stability into proteins by site-directed mutagenesis [14].

*Dm*AChE is a dimer linked to membrane via a GPI anchor. There are eight cysteines in each monomer [15]. Six are involved in intrachain disulfide bonds, they are highly conserved in the protein family and their mutations result in inactivation of the protein. One cysteine is involved in an interchain disulfide bond and one, at position 290 (328 using precursor numbering) remains free [16,17]. The aim of this work was to stabilize *Dm*AChE by introducing new disulfide bonds.

## Results

### Mutation

There are 35 potential disulfide bridges in *Dm*AChE if we consider that every distance between two Cβ of 3.6 to 4 Å is suitable to form a disulfide bridge following the mutation of the two residues in cysteines. Among them, we selected 7 using two criteria: the two amino-acids involved should not be conserved in the cholinesterase family and a serine at these positions is present in one of the available sequences [18]. All these 7 disulfide bonds were predicted by MODIP, automated software for modeling disulfide bonds in proteins [19] with grades A (ideal stereochemistry), B (geometrically suitable but with distorted stereochemistry) and C (sites close enough to allow the formation of a disulfide bond) [20]. We verified that the engineered disulfide bonds were formed by assaying free sulfhydryl groups with the Ellman reagent in the presence of 6 M urea. The results were consistent with the expected disulfide bonds. We verified that the new cysteines did not promote a higher degree of polymerisation. SDS-gel electrophoresis performed in non-reducing conditions showed that all mutants were dimeric proteins like the wild type: introduction of cysteines did not provide additional intersubunit interactions in the mutants.

In our conditions, production of wild type *Dm*AChE in insect cells via the secretory network is 52 nmoles per liter, five bridges did not significantly affect this protein production; two, m3 and m4 decreased production and no mutation increased production (Table 1).

**Table 1:** Production ratio of mutant in baculovirus compared to wild type enzyme. Reference is the wild type *Dm*AChE which was produced at 52 nanomoles per liter of culture. *: significant difference, n: number of batches analyzed

				Relative production		
Mutant code	Mutated amino-acids	Grade (MODIP)	distance (Cβ) in tertiary structure (Å)	n	mean	Standard error

m1	R24/A169	B	3.65	5	1.43	0.71
m2	I327/D375	C	3.77	20	1.06	0.73
m3	L354/A456	C	3.56	19	0.22 *	0.26
m4	T369/M476	A	3.62	15	0.04 *	0.07
m5	L388/Q427	C	3.70	16	0.96	0.63
m6	A452/S533	B	3.91	7	0.73	0.45
m7	T464/S543	D	3.96	8	0.77	0.47

### Heat denaturation

**Figure 1:**
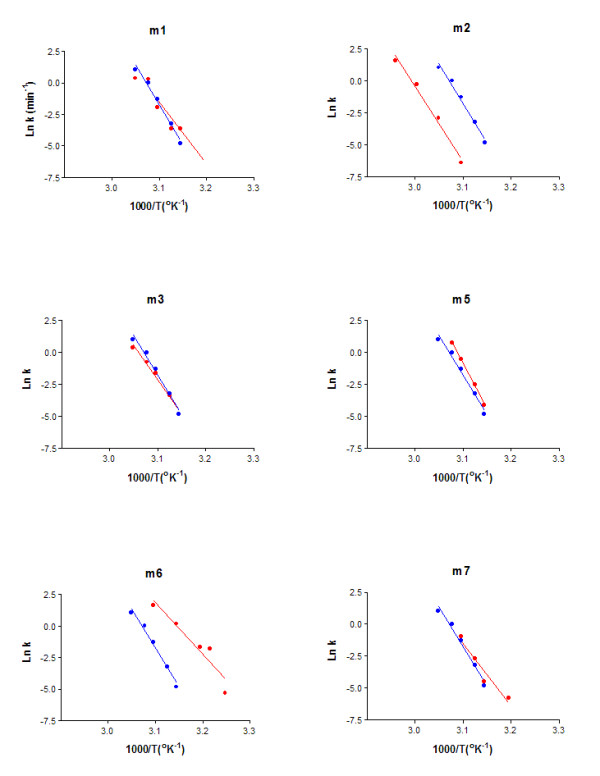
Arrhenius plots of thermal inactivation rate constants of mutated *Dm*AChE (in red) compared to wild type (in blue). *k*: denaturation first order rate constant (in min^-1^).

We first analyzed denaturation with the most common method used to study protein denaturation: incubation at high temperature. The stability of the mutated protein was estimated by studying irreversible thermal inactivation at several temperatures (from 35 to 65°C) and plotted the first-order denaturation rate constant (*kd*) against the reciprocal of the absolute temperature (°K^-1^). It appeared that one bridge (m2) increased thermostability while one (m6) decreased it (Fig. 1).

### Urea and organic solvent denaturation, protease sensitivity

Stability was assayed with three denaturing agents. In all cases, denaturation was irreversible and followed apparent first order kinetics. Stability was characterized by the half-life (*t*_50_), the time at which 50% of an initial enzymatic activity is preserved. The half life of the wild type protein was 13.6 min. in 4 M urea. Protease was used as a denaturant because a protein's resistance to proteolysis increases with its conformational stability due to the fact that the susceptibly to proteolysis reflects the rate of local unfolding [21,22]. The half life of wild type *Dm*AChE was 13.9 min in 0.1 mg/ml pronase. Detection of insecticides in food requires their extraction with organic solvent. Although the solvent should be eliminated before the assay, low amounts may remain in solution and inactivate the enzyme. We used acetonitrile as model because it is soluble in water. The half life of the wild type protein was 1.7 min in 20% acetonitrile. The thermostability provided by bridge m2 is conserved for other denaturing agents (Table 2). Identically, the low stability provided by bridge m6 is found again. In addition, low stability was found for bridges m1 and m5.

**Table 2:** Relative stability of mutated AChEs. For each mutation, the t50 ratio (t50 mutant/t50 wild type) was calculated for each denaturation agent (*: significant difference. n: number of independent batches analyzed)

mutation	n	50°C	20% acetonitrile	4 M urea.	0.1 mg/mL pronase.
m1	1	2	0.43*	0.40*	0.27*
m2	10	170*	2.11*	12.35*	1.60*
m3	9	1.4	0.85	1.87*	2.37*
m5	6	0.47*	0.13*	0.05*	0.17*
m6	2	0.05*	0.33*	0.26*	0.71
m7	2	0.73	0.94	0.71	0.79

### Specific activity

**Figure 2:**
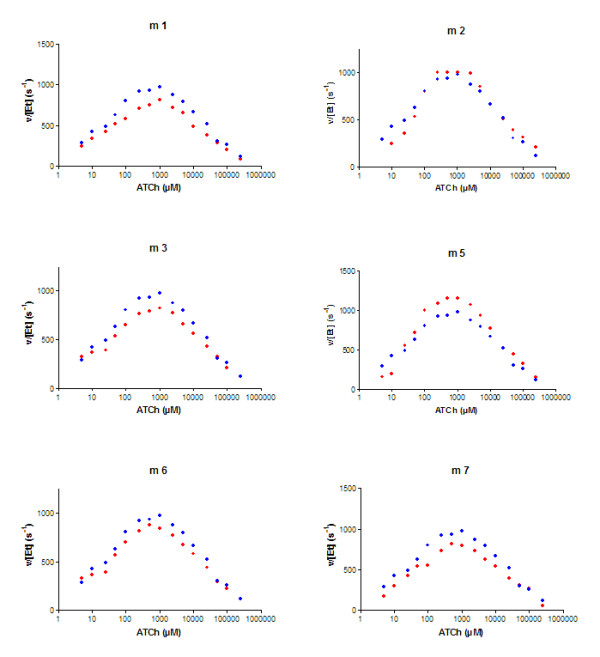
**Effect of mutations on acetylthiocholine hydrolysis versus substrate concentration (log scale)**. (blue dots): wild type; (red dots): mutant, Acetylthiocholine concentration in micromoles per liter; v/[Et] specific activity in s^-1^.

The specific activity of the mutants, and the patterns of the pS curves, were not significantly changed with the introduction of new bridges (Fig. 2). This suggests that entrance of the substrate into the active site as well as the catalytic efficiency was not affected by the mutations.

## Discussion

From the first works of Villafranca et al. [23] and Perry and Wetzel [24], introduction of non-native disufide bonds has been used to stabilize proteins [25-34]. These successes pushed us to use this technique to stabilize *Dm*AChE.

### The effect of addition of disulfide bridges was either stabilization or destabilization

Most new disulfide bonds introduced in *Dm*AChE did not affect protein stability, one decreased stability. Destabilization has sometimes been reported [35,36]. This instability has been interpreted as the result of atypical sets of dihedral angles in newly formed disulfide bridges [37], from stabilization of the denatured state [38] or from reduction of disulfide bonds followed by disulfide exchange or chemical reaction of the SH groups formed [39,40]. Attempts to predict destabilization by modeling using MODIP failed, suggesting that selected positions were too flexible for a fulfilling prediction.

**Figure 3:**
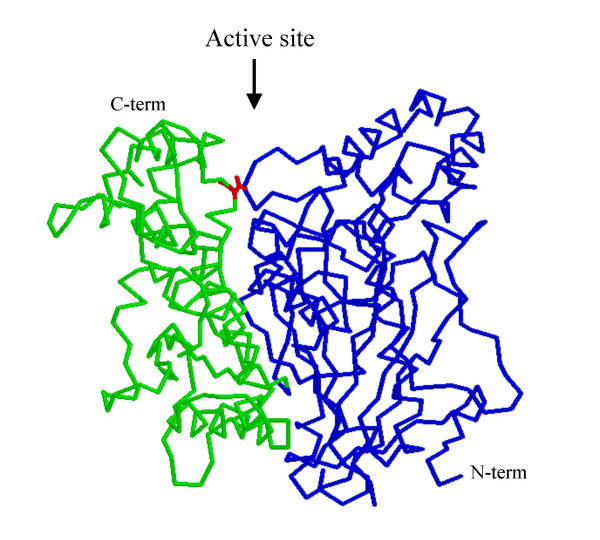
**Position of mutation m2 (I327/D375)**. The cross link has been colored in red. The disulfide bridge links two subdomains of the protein.

We found one mutation which stabilizes the protein (m2). Two subdomains forming the active site may be distinguished in cholinesterases and mutations decreasing interactions between them decrease protein stability [41]. Disulfide bridge m2 links the two subdomains of the enzyme (Fig. 3), strengthens subdomain interactions and increases overall stability. This suggests that the contact area of the two subdomains is the weakest site of the protein, taking into account the hypothesis that unfolding of a protein molecule starts at its weakest site, and local stabilization of this fragile region results in global stabilization of the whole molecule [42].

### Addition of new disulfide bonds may impair protein production

Production is a key issue for application of the stable enzymes in biosensors. We found that addition of a disulfide bond may result in a decrease of protein production since two mutations out of the seven studied, affected protein production. Most probably, increasing the number of sulfhydryl groups in a protein decreases the folding efficiency by increasing the number undesirable disulfide bonds which results in a misfolded protein.

## Conclusion

Addition of a disulfide bridge may either stabilize or unstabilize proteins.

## Methods

### Protein engineering

Possible sites for the introduction of disulfide bonds were located according to Wakarchuk *et al*. [23], by searching for pairs of residues for which the inter- Cβ distance was between 3.6 and 4 Å in the structure of *Dm*AChE [17].

### Protein production and purification

cDNA encoding *Dm*AChE and mutants were expressed with the baculovirus system [43]. We expressed a soluble dimeric form deleted of the hydrophobic peptide at the C-terminal end which is exchanged for a glycolipid anchor. A 3 × histidine tag replaced the loop from amino-acids 103 to 136 to facilitate purification. This external loop is at the other side of the molecule with respect to the active site entrance and its deletion affects neither the activity nor the stability of the enzyme. Secreted AChE was purified to homogeneity using the following steps, ammonium sulfate precipitation, ultrafiltration with a 50 kDa cut off membrane, affinity chromatography with procainamide as ligand, NTA-nickel chromatography and anion exchange chromatography [7]. Residue numbering followed that of the mature protein.

### Enzyme activity

The kinetics of substrate hydrolysis was followed at 25°C in 25 mM sodium phosphate buffer pH 7, containing 1 mg/ml BSA. Hydrolysis of acetylthiocholine, an analogue of the neurotransmitter allowing easy detection of the reaction product, was studied spectrophotometrically at 412 nm using the method of Ellman *et al*. [44], at substrate concentrations ranging from 2 μM to 300 mM, in 1 cm path-length cuvettes. Activity was measured for 1 minute after addition of the enzyme to the reaction mixture. The concentration of the enzymes was determined by active site titration using irreversible inhibitors with high affinity [45].

### Denaturation

*Dm*AChE is denatured irreversibly, ΔG_d _cannot be determined. Instead, the changes in the stability relative to a wild-type protein may be defined as the rate of enzymatic activity decrease [46]. All denaturation experiments were performed with 10 picomoles enzyme in 1 ml 25 mM phosphate buffer pH7 at 25°C. AChE was incubated in denaturing conditions, aliquots were taken out at regular times, diluted 10-fold in enzyme reaction mixture and remaining activity was measured, since residual enzymatic activity is related to the proportion of non-denatured protein. To analyze heat sensitivity, enzymes were incubated at 50°C and 1 mg/ml bovine serum albumin was added to the buffer. Aliquots were mixed with cold buffer chilled on ice and the solution was incubated at 25°C for ten minutes before recording the remaining activity. For urea denaturation, unfolding of *Dm*AChE was induced by adding 4 M urea to the incubation buffer. The effect of organic solvent was followed by incubation of the enzyme in 20% acetonitrile. The effect of protease sensitivity was determined by incubation of AChE with 0.1 mg/ml pronase.

## Abbreviations

*Dm*AChE: Drosophila acetylcholinesterase, ATCh: acetylthiocoline, BSA Bovine Serum Albumin.

## Authors' contributions

AL and LL performed *in vitro *mutagenesis, ORS and CL produced the protein and performed biochemical analysis. DF conceived and coordinated the study. All authors participated in the interpretation of the results, in the writing and revising of the manuscript, read and approved the final manuscript. This work has been supported by grants from CRSSA (Centre de recherche du Service de Santé de l'Armée) and the European contract n° QLK3-CT-2000-00650.
